# Predictors of hydrocephalus as a complication of non-traumatic subarachnoid hemorrhage: a retrospective observational cohort study in 107 patients

**DOI:** 10.1186/s13037-018-0160-6

**Published:** 2018-05-22

**Authors:** Juan Manuel Vinas Rios, Martin Sanchez-Aguilar, Thomas Kretschmer, Christian Heinen, Fatima Azucena Medina Govea, Sanchez-Rodriguez Jose Juan, Thomas Schmidt

**Affiliations:** 1 Sana Spine center, Klinikum Offenbach, Bruchfeld Str. 74, 60528 Offenbach, Germany; 2 Clinic epidemiology, Universidad Autonoma de San Luis Potosi, San Luis Potosí, Mexico; 3 Department of neurosurgery, Klinikum Klagenfurt, Klagefurt, Austria; 4 Department of neurosurgery, University clinic Evangelical Hospital Oldenburg, Oldenburg, Germany; 5 Department of neurosurgery Klinikum Braunschweig, Braunschweig, Germany

**Keywords:** Hydrocephalus, Shunt dependency, Subarachnoid hemorraghe, Vasospasmus, Intraventricular hemorraghe

## Abstract

**Background:**

The predictors of shunt dependency such as amount of subarachnoid blood, acute hydrocephalus (HC), mode of aneurysm repair, clinical grade at admission and cerebro spinal fluid (CSF) drainage in excess of 1500 ml during the 1st week after the subarachnoid hemorrhage (SAH) have been identified as predictors of shunt dependency. Therefore our main objective is to identify predictors of CSF shunt dependency following non-traumatic subarachnoid hemorrhage.

**Methods:**

We performed a retrospective study including patients from January 1st 2012 to September 30th 2014 between 16 and 89 years old and had a non-traumatic subarachnoid hemorrhage in cranial computed tomography (CCT). We excluded patients with the following characteristics: Patients who died 3 days after admittance, lesions in brainstem, previous surgical treatment in another clinic, traumatic brain injury, pregnancy and disability prior to SAH.

We performed a descriptive and comparative analysis as well as a logistic regression with the variables that showed a significant difference (*p* < 0.05). Hence we identified the variables concerning HC after non traumatic SAH and its correlation.

**Results:**

One hundred and seven clinical files of patients with non-traumatic SAH were analyzed. Twenty one (48%) later underwent shunt treatment. Shunt patients had significantly clinical and corroborated with doppler ultrasonography vasospasmus (*p* = 0.015), OR = 5.2. The amount of subarachnoidal blood according to modified Fisher grade was (*p* = 0.008) OR = 10.9. Endovascularly treated patients were less often shunted as compared with those undergoing surgical aneurysm repair (*p* = 0.004).

**Conclusion:**

Vasospasmus and a large amount of ventricular blood seem to be a predictor concerning hydrocephalus after non-traumatic SAH. Hence according to our results the presence of these two variables could alert the treating physician in the decision whether an early shunt implantation < 7 days after SAH should be necessary.

## Background

Spontaneous subarachnoid hemorrhage (SAH) is one of the most common disorders within the vast neurological field involving morbidity-mortality. The mean age of presentation is at the age of 55 years, affecting younger people that the rest of the cerebral insults such as cerebral ischemia [1].

Hydrocephalus (HC) is a frequent complication of SAH. While acute HC is present in 15-87% [2–6], chronic HC has been reported in 9-64% of SAH [7–13].

Acute HC requires urgently cerebrospinal fluid (CSF) drainage in order to reduce the intracranial pressure (ICP) and to avoid cerebral herniation. Early CSF drainage has been proven to be beneficial in reducing secondary effects from SAH. Shunt surgery carries risks of complications such as cerebral bleeds, subdural hematoma and infection [14–18]. The predictors of shunt dependency such as amount of subarachnoid blood, acute HC, mode of aneurysm repair, clinical grade at admission and CSF drainage in excess of 1500 ml during the 1st week after the ictus have been identified as predictors of shunt dependency. Nevertheless there are discrepancies in these predictor factors about their influence in patients being shunted [3, 7, 9, 12, 13, 19, 20]. Taking in count these discrepancies our main objective was to confirm and/or to reveal, until now, not taken in count predictor factors in shunt dependency following spontaneous subarachnoid hemorrhage.

## Methods

We developed a retrospective study. We evaluated clinical profiles of 107 patients included from January 1st 2012 to September 30th 2014. We compared shunted and non-shunted patients with regard age, gender, clinical grade at admission (Hunt and Hess grade), aneurysm location, amount of subarachnoid blood (modified Fisher grade), amount of interventricular blood (LeRoux), and ventricular size (Evans Index, third ventricle index and ventricular score) on preoperative cranial computer tomography (CCT). We further compared the treatment modality, surgical opening of the lamina terminalis, decompressive craniectomy, use of ventricular/Lumbar CSF drains, and volume of CSF drained during week 1 and weeks 1-3 in the two patients groups (shunted vs. non-shunted patients).

Inclusion Criteria:

- Patients between 16 and 89 years old
- Non-traumatic subarachnoid hemorrhage in cranial computed tomography (CCT)

The evaluated patients were included from the registries from the University Clinic Evangelisches Krankenhaus, Oldenburg, Germany. Twenty patients that did not fulfill the inclusion criteria were excluded.

The present study was carried out in a high-volume over-regional neurosurgical center managing approximately 100 non-traumatic SAH patients yearly from a defined catchment area where the national government covers all medical expenses. To limit other biases with regard to defining HC after spontaneous SAH, we simply defined shunt dependency as having received a shunt.

Exclusion criteria:

- Patients who died 3 days after admittance.
- Lesions in the intraparenchymatous brainstem as an isolated finding.
- Previous surgical treatment in another clinic.
- Pregnancy.
- Prior disability to SAH.
- Traumatic brain injury

### Management and Intervention

The patients were admitted to the emergency room and handled according to the guidelines of Advanced Trauma Life Support (ATLS). Once the patients were stabilized CCT scan were taken.

The routine treatment included a crystalloid solution, gastric protector, analgesic and sedative in case of agitation. For intubation purposes we used propofol plus rocuronium.

At the admittance we performed a native CCT and CT-angiography, if negative a four vessels 3D Digital Substraction Angiographie (DSA) was obtained; if this still remains negative an MRI plus MRI-angiography was done. After negative results for an aneurysm bleeding in MRI plus MRA-angiography, we admitted the patient in the ICU performing Intracranial Doppler Ultrasonography. If Vasospasm was diagnosticated, the Intracranial Doppler Ultrasonography was performed every 24 h. If the DSA and the MRI plus MRI- angiography was negative, the SAH was classified as spontaneous prepontine SAH and was treated conservatively.

We defined patients receiving a shunt as shunt depending patients. The decision for shunting was based on our institutional treatment algorithm.

The radiological evaluation of the amount of subarachnoid and ventricular blood, as well as ventricular size, was performed retrospectively. The amount of SAH was evaluated using the modified Fisher grade. We further differentiated between supra- and infratentorial bleeds. For linear measures of ventricular size, we determinate the Evans index, Third ventricle index and ventricular score. The LeRoux scoring system was used for grading the amount of interventricular blood.

All the patients were managed with the goal of obtaining a mean ICP threshold of less than 20 mmHg.

### Statistical analysis

We utilized the program JMP-7. We completed an analysis of descriptive statistics, obtaining the measures of central tendency and dispersion of all the variables. For the comparative analysis we used the Student’s t-test for continuous variables with normal distribution and the Wilcoxon/Kruskal-Wallis test for continuous variables without normal distribution. For categorical variables the Chi-squared test was applied and for tables with boxes less than 5, the Fisher’s exact test was utilized. Statistical significance was considered with a value of *p* < 0.05. We calculated the Odds Ratio (OR) with of Confidence Intervals (CI) of 95%. We made an analysis of logistic regression with the variables that showed a significant difference (*p* < 0.05) in the bivariate analysis. In the final model, they were expressed with OR (CI 95%), as well as with multiple coefficient correlation R2.

## Results

In our study almost the half of the patients (41%) received an external ventricle or lumbar CSF drains (EVD/LD). From these 44/107 patients (41%), 21 (48%) later underwent a permanent CSF drain. They were shunted with a median of 15.3 days (12.5-17.7) after bleed. The elected treatment of the patients with a proven aneurysm was: 40 endovascular, 39 with surgical clipping and 28 were treated conservative as prepontine SAH. Two patients (1.8%) underwent shunt treatment without previously EVD/LD placement.

The aneurysm location was not significantly different between the shunt and non-shunt groups.

The amount of subarachnoid blood according to modified Fisher grade as presented in Table 1 was (*p* = 0.008). While the non-shunted group had a larger fraction of patients with minimal SAH, shunted patients had a higher fraction of patients with large SAH. Moreover, shunted patients had significantly more intraventricular blood (LeRoux 4 or more, Table 1).

**Table 1:** Demographic, clinical, and radiological variables at admission and early management of the studied patients

	Shunt (*n* = 21)	Non-Shunt (*n* = 86)	*p*-Value
Gender (M/F)	6/15	42/44	0.53
Age (years) (median, range)	57 (29-78)	56 (21-83)	0.77
Clinical grade at admission			
H-H^a^ 1-3	15 (71.4%)	65 (75.5%)	0.72
H-H^a^ 4-5	6 (28.6%)	21 (24.5%)	
Radiology at admission			
Modified Fisher 1 + 2 (minimal SAH)	3 (14.2%)	38 (44.2%)	
Modified Fisher 3 + 4 (large SAH)	18 (85.8%)	48 (55.8%)	0.008
Bleeding Supratentorial	5 (24%)	18 (21%)	
Bleeding Infratentorial	1 (5%)	0	0.09
Evans index (median, range)	0.29 (0.23-0.33)	0.27 (0.21-0.36)	0.52
Third ventricle in mm	8 (4.7-13.7)	6.9 (3-14.3)	
Ventricular Score	79.3 (60-92.6)	70 (51-103.7)	0.003
Aneurysmal Source of bleeding			
Middle cerebral artery	7 (33%)	20 (23%)	
Internal carotid artery	3 (14%)	8 (9%)	
Anterior cerebral artery	5 (24%)	21 (24%)	
Posterior circulation	4 (19%)	11 (13%)	
Non-aneurysmal	2 (9%)	26 (30%)	0.08
Surgical Intervention			
EVD/LD^b^	16 (76%)	28 (32%)	< 0.001
Lamina Terminalis Opening	5 (24%)	17 (20%)	0.09
Decompressive craniectomy	1 (5%)	6 (7%)	0.07
CSF Drainage			
Drainage week 1 (ml) (median, range)	1523 ± 158	1258 ± 122	0.63
Drainage week 1-3 (ml) (median, range)	3364 ± 308	2683 ± 233	0.76
Clinical vasospasm	15 (71%)	36 (42%)	0.015

Significant differences between groups were determined by chi-square test or Fisher exact test for dichotomized or categorical data as appropriate

Continuous data were determined using independent sample T-student test or Mann-Whitney U-test

^a^ HH Hunt and Hess grade

^b^ EVD/LD Extraventricular drainage or lumbar drainage performed

**Fig. 1:**
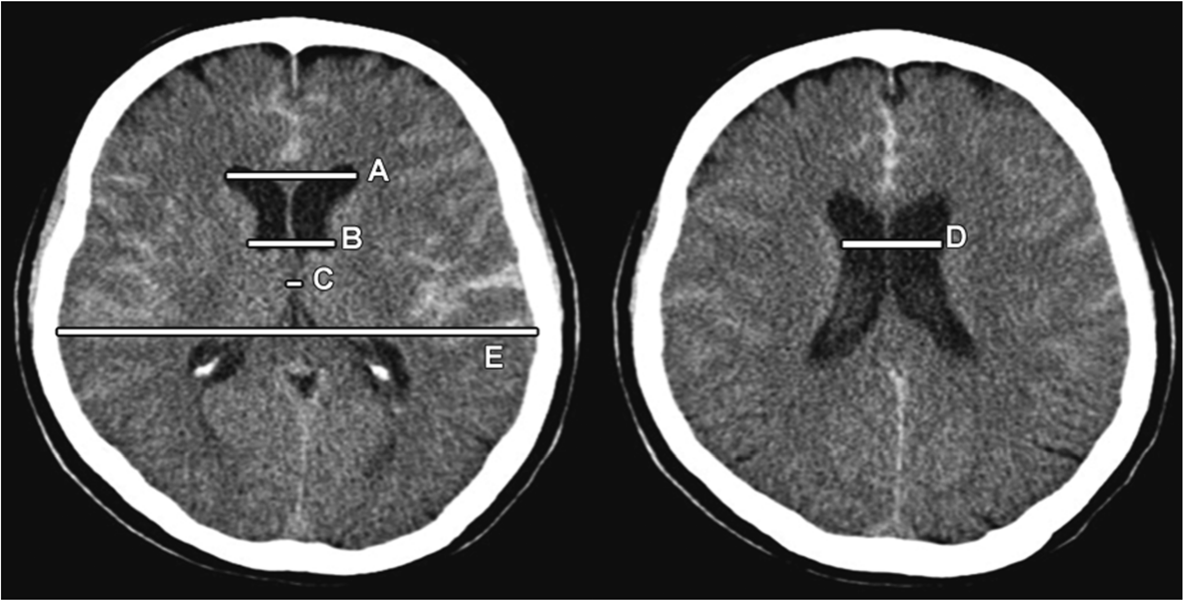
Example of ventricle size estimation

The linear measures of ventricular size also differed significantly between groups with shunt patients having significantly larger ventricles in terms of ventricular score at admission compared to the non-shunted patients (*p* = 0.003) (see Fig. 1).

Endovascular treated patients were less often shunted as compared with those undergoing surgical aneurysm repair (20% vs 26%, *p* = 0.004). Moreover EVD/LD and decompressive craniectomy were significant between the treatment groups (60% vs 36%, *p* = 0.03 and 0 vs 18%, *p* = 0.001 respectively). The surgical opening of the lamina terminalis was not significant between the groups. From the patients with prepontine SAH, treated conservatively 3 (14%) received a shunt (not shown).

Shunted patients had not significantly higher volumes of CSF drainage during week 1 and week 1-3 as compared to those that were not shunted.

The endovascular treated patients had a higher ventricular score at the admittance 71.5 (58-94.7) vs 70.2 (66.8-73.6), *p* < 0.001. There was a larger amount of intraventricular blood in patients who were treated endovascular. The mode of aneurysm repair was closely linked to aneurysm location (*p* < 0.001); thus, while 21(54%) patients that bled from middle cerebral artery aneurysms underwent surgical repair, 11 of the 15 patients with vertebrobasilar artery aneurysm bleeds were treated endovascular (Table 2).

**Table 2:** Endovascular treatment (EVT) versus surgical treatment (clipping) in the studied patients

	EVT (*n* = 40)	Surgical (*n* = 39)	p-Value
Gender (M/F)	16/24	13/26	0.75
Age (years)	53 (21-80)	55 (23-77)	0.87
Clinical grade at admission H-H			
H-H^a^ 1-3	27 (67.5%)	25 (64%)	0.08
H-H^a^ 4-5	13 (32.5%)	14 (36%)	
Radiology At Admission			
Modified Fisher 1 + 2 (minimal SAH)	12 (30%)	8 (20.5%)	0.08
Modified Fisher 3 + 4 (large SAH)	28 (70%)	31 (79.5%)	
Bleeding Supratentorial	6 (15%)	17 (43%)	
Bleeding Infratentorial	0	1 (2%)	0.006
Evans index	0.28 (0.22-0.34)	0.27 (0.21-0.33)	0.45
Ventricular Score	71.5 (58-94.7)	70.2 (66.8-73.6)	0.001
Le Roux 0-3	0	7 (18%)	0.001
Le Roux ≥4	40 (100%)	32 (82%)	
Aneurysmal source of bleeding			
Middle cerebral artery	6 (40%)	21 (54%)	
Internal carotid artery	8 (20%)	3 (8%)	
Anterior cerebral artery	15 (37%)	11 (28%)	
Posterior circulation	11 (27%)	4 (11%)	0.001
Surgical intervention			
Shunt	8 (20%)	10 (26%)	0.004
EVD/LD	24 (60%)	14 (36%)	0.03
Lamina Terminalis Opening	N/A	22 (56%)	
Decompressive craniotomy	0	7 (18%)	0.001
CSF Drainage			
Drainage week 1 (ml) (median, range)	1427 ± 456	1495 ± 772	0.72
Drainage week 1-3 (ml) (median, range)	2635 ± 1020	3107 ± 1240	0.67
Clinical Vasospasmus	27 (67%)	16 (41%)	0.023

Significant differences between groups are determined by Pearson chi-square test. Continuous data were determined using the independent sample t test

^a^ HH Hunt and Hess grade

^b^ EVD/LD Extraventricular drainage or lumbar drainage performed

The volume of CSF drained during both week 1 and weeks 1-3, age, gender, preoperative Hunt and Hess grade and amount of subarachnoid blood did not differ significantly between patients undergoing surgical versus endovascular aneurysm repair.

In the univariable analysis (Table 3), vasospasm was a strong predictor with an odds ratio (OR) of 5.2 for shunt dependency when compared with patients that did not develop this condition. Larger amounts of intraventricular blood increased the likelihood of shunt dependency, with ORs of 10.9 for intraventricular blood with LeRoux score ≥ 4.

**Table 3:** Best logistic regression model for shunt dependency

Parameter	OR	Lower 95%	Upper 95%	*p* value
Vasospasmus	5.7	4.7	6.7	0.016
Intraventricular Hemorrhage (LeRoux ≥4)	10.9	8.0	12.8	< 0.001

## Discussion

The main findings of this study were that shunt dependency after spontaneous SAH could be predicted by larger amounts of subarachnoid and ventricular blood or large ventricular size being high on admission CT scans, clinical and doppler ultrasonographic vasospasm and the colocation of a drainage system (EVD/LD). Meaning that patients with large amounts of intraventricular blood in the CT on admission (LeRoux score ≥ 4) with EVD/LD colocation because of acute hydrocephalus and consequent vasospasm because of the large amount of blood with intracerebral vessels irritation/inflammation and adhesion in the ventricular and subarachnoid space causing CSF circulation disturbances.

### Patient material

Our patient material consisted of 107 retrospective spontaneous SAH patients including those with non-aneurysm finding. The patients received their shunt either after unsuccessful weaning of a temporary CSF drain during the primary stay or, less common during re-hospitalization at our department due to secondary clinical deterioration combined with increasing ventricular size.

Median time from ictus to shunting was 15 days. The mode of weaning of temporary CSF drainage does not seem to influence the frequency of shunt dependency 10, and our treatment protocol should hence not be crucial to the observed results. A weakness of the present study was the limited number of patients investigated and the retrospective data collection.

### Treatment algorithm for SAH patients

The patients included followed this department’s routine for management of SAH patients. In short, our policy includes immediate identification of the origin of hemorrhage, early aneurysm repair, and rigorous prevention of secondary brain damage.

Rigorous CSF drainage is used during the early period. In patients with evidence of shunt dependency, we advocate early shunt implantation.

### Assessment and treatment of chronic hydrocephalus after SAH

Assessment of chronic HC after spontaneous SAH is a challenge. Actually, since there is no single test to determine the presence or absence of shunt-dependent HC, it becomes difficult to determine the frequency of chronic HC after spontaneous SAH and therefore a shunt system implantation. This could partly be the reason why the literature reports frequencies of chronic HC ranging from 9 to 64% after spontaneous SAH [3, 8, 9, 11, 12]. In this study, 20% of the patients were shunted, a number according within mentioned reports.

### Shunt dependency versus clinical, radiological and aneurysmal variables

Many studies have found that acute HC predicts shunt dependency/chronic HC [3, 8, 19, 21, 22]. It has been proven that clinical grade at admission, a larger amount of subarachnoid blood and large ventricular size on preoperative cerebral CT, and particularly CSF drainage in excess of 1500 ml during the 1st week after the ictus were significant predictors of shunt dependency [23].

In our study the shunted patients had larger cerebral ventricles on CT scans at admission than the non-shunted patients, i.e., the ventricular score was a significant predictor of shunt dependency.

As observed by others and in the present study, the amount and distribution of subarachnoid blood is, per se, a significant predictor of later shunt dependency after SAH [5]. This blood then interacts with tissue and leads to disturbed CSF circulation [6]. On the other hand, hydrocephalus has been discovered as a complication of spontaneous subarachnoid hemorrhaging, manifesting in ventricular hemorrhaging, vasospasm, and aneurysms of the posterior segment as well as damage due to intraoperative manipulation [24, 25]. This last asseveration correlate with our finding that patients treated with surgical clipping underwent more shunt system colocation in contrast with the patients treated with coiling. This could be explained because of the intraoperative manipulation with consequent induction of vasospasm; thus disturbed CSF circulation could be due to fibrosis of the subarachnoid space.

In our univariable logistic regression analysis, we found clinical and doppler ultrasonographic vasospasmus to be a predictor of shunt dependency. Intraventricular hemorrhage hence increased the likelihood of developing shunt dependency. This concurs with findings by others [6, 9, 22, 26].

Most vertebrobasilar aneurysms were treated endovascular in our study. This could explain the finding that aneurysm location was not a predictor of shunt dependency in the multivariable analysis.

### Shunt dependency versus treatment variables

In our multivariable analysis we found that clinical and doppler ultrasonographic vasospasm together with intraventricular blood at admittance were the strongest predictor of later shunt dependency; vasospasm hence increased the likelihood to become shunted almost 5 times and intraventricular blood 10.9 times.

Recent experimental studies have demonstrated abnormally increased CSF production following spontaneous SAH, which might be an important pathophysiological mechanism behind acute HC after spontaneous SAH [26].

The effect of the mode of aneurysm repair on shunt dependency has been discussed in the literature [15, 17, 27]. It has been concluded that there is less risk for developing shunt-dependent HC after surgical clipping as compared to endovascular aneurysm repair [27].

Although prepontine SAH is considered a bleeding with a better prognostic in comparison with aneurysm bleeding [28], it has been described as producing hydrocephalus in a mild number of patients suffering this condition as seen in our study. A predictor factor in developing chronic hydrocephalus after prepontine SAH is a heavy clot burden (Fisher grade 3) [29].

In the multivariable logistic regression analysis, however, mode of aneurysm repair was not a significant predictor. The effect of surgical opening of the lamina terminalis on the development of chronic HC has been debated. Whereas the guidelines for treatment of spontaneous SAH patients do not recommend opening of the lamina terminalis [2, 30], however it has been found lamina terminalis fenestration to be effective in preventing chronic HC. Our findings do not support this notion.

Many of the previously published predictive factors for shunt dependency are interwoven. Presently, the multivariable logistic regression analysis is helpful in sorting out which predictors are relevant per se. The data of this study suggest that patients with a large amount of intraventricular blood at admission and patients developing clinical and doppler ultrasonographic vasospasm should be considered for shunt placement as soon as possible.

As shortcoming in our study, based in our result, the following asseverations could be assessed:

- Patients suffering vasospasmus in the 1st week after Hemorrhage have a higher risk to develop hydrocephalus.
- Large amounts of intraventricular blood after SAH is the most significant factor in developing post-hemorrhagic hydrocephalus.
- The amount of drained CSF and localization of the aneurysm, after SAH does not play a role in developing hydrocephalus.
- At the beginning of our study seemed to be clipping aneurysm repair a risk factor for developing hydrocephalus. After the logistic regression analysis it was discharged as a variable for outcome regarding post-hemorrhagic hydrocephalus.

## Conclusions

In this retrospective study, vasospasmus and a large amount of ventricular blood seem to be a predictor concerning hydrocephalus after non-traumatic SAH. Hence the presence of these two variables could alert the treating physician in the decision whether an early shunt implantation < 7 days after SAH should be necessary. For a more accurate representation of this phenomenon, prospective studies with a larger amount of patients and a longer follow up in shunted patients with post-hemorrhagic hydrocephalus should be performed.
